# The FibromiR miR-214-3p Is Upregulated in Duchenne Muscular Dystrophy and Promotes Differentiation of Human Fibro-Adipogenic Muscle Progenitors

**DOI:** 10.3390/cells10071832

**Published:** 2021-07-20

**Authors:** Nicole Arrighi, Claudine Moratal, Grégoire Savary, Julien Fassy, Nicolas Nottet, Nicolas Pons, Noémie Clément, Sandy Fellah, Romain Larrue, Virginie Magnone, Kevin Lebrigand, Nicolas Pottier, Claude Dechesne, Georges Vassaux, Christian Dani, Pascal Peraldi, Bernard Mari

**Affiliations:** 1 Faculté de Médecine, CNRS, Inserm, iBV, Université Côte d’Azur, 06107 Nice, France; Nicole.ARRIGHI@univ-cotedazur.fr (N.A.); Claudine.MORATAL@univ-cotedazur.fr (C.M.); noemiec06@yahoo.fr (N.C.); cl.dechesne@gmail.com (C.D.); Pascal.Peraldi@univ-cotedazur.fr (P.P.); 2 FHU-OncoAge, CNRS, IPMC, Université Côte d’Azur, 06560 Valbonne, France; gregoire.savary@ibl.cnrs.fr (G.S.); fassy@ipmc.cnrs.fr (J.F.); nicolas.nottet@gmail.com (N.N.); nicolaspons4@gmail.com (N.P.); magnone@ipmc.cnrs.fr (V.M.); lebrigand@ipmc.cnrs.fr (K.L.); vassaux@ipmc.cnrs.fr (G.V.); 3 CANTHER-Cancer Heterogeneity Plasticity and Resistance to Therapies, Institut Pasteur Lille, CNRS, Inserm, CHU Lille, Université de Lille, UMR9020-UMR-S 1277, 59000 Lille, France; sandy.fellah@inserm.fr (S.F.); romain.larrue@univ-lille.fr (R.L.); nico_pottier@yahoo.fr (N.P.)

**Keywords:** microRNA (miRNA), transforming growth factor beta (TGFβ), fibrosis, fibro-adipogenic progenitor (FAP), Duchenne muscular dystrophy (DMD), FGFR1

## Abstract

Fibrosis is a deleterious invasion of tissues associated with many pathological conditions, such as Duchenne muscular dystrophy (DMD) for which no cure is at present available for its prevention or its treatment. Fibro-adipogenic progenitors (FAPs) are resident cells in the human skeletal muscle and can differentiate into myofibroblasts, which represent the key cell population responsible for fibrosis. In this study, we delineated the pool of microRNAs (miRNAs) that are specifically modulated by TGFβ1 in FAPs versus myogenic progenitors (MPs) by a global miRNome analysis. A subset of candidates, including several “FibromiRs”, was found differentially expressed between FAPs and MPs and was also deregulated in DMD versus healthy biopsies. Among them, the expression of the TGFβ1-induced miR-199a~214 cluster was strongly correlated with the fibrotic score in DMD biopsies. Loss-of-function experiments in FAPs indicated that a miR-214-3p inhibitor efficiently blocked expression of fibrogenic markers in both basal conditions and following TGFβ1 stimulation. We found that FGFR1 is a functional target of miR-214-3p, preventing the signaling of the anti-fibrotic FGF2 pathway during FAP fibrogenesis. Overall, our work demonstrates that the « FibromiR » miR-214-3p is a key activator of FAP fibrogenesis by modulating the FGF2/FGFR1/TGFβ axis, opening new avenues for the treatment of DMD.

## 1. Introduction

Fibrosis is a deleterious invasion of tissues associated with many pathological conditions such as idiopathic pulmonary fibrosis and tubulointerstitial kidney fibrosis [1]. It corresponds to the disproportionate synthesis of the extracellular matrix (ECM), with high accumulation of specific proteins, including collagen, fibronectin, connective tissue growth factor and matrix metalloproteinases [2]. Most tissues can be affected, usually as a result of repeated injuries or chronic inflammation. Fibrosis modifies the structure of the resident tissue and affects the overall homeostasis of the organ, leading, in extreme cases, to the loss of functionality of the organ affected.

The cell type responsible for fibrosis is myofibroblast. Myofibroblasts are specialized fibroblasts that contain stress fibers expressing α-smooth muscle actin (α-SMA), which confers contractility. They produce abundant ECM and secrete various growth factors. Myofibroblasts appear during wound healing and are normally de-activated or disappear through apoptosis after restoration of healthy tissue organization [3]. Fibrosis is due to the pathological persistence of myofibroblasts. Myofibroblast differentiation is regulated by several signaling pathways among which TGF-β is recognized as a main inducer [4].

Due to its involvement in myopathies, muscle fibrosis is particularly studied. In striated muscles, fibro-adipogenic degeneration is commonly found in several pathophysiological situations affecting cardiac and skeletal muscles, including aging and muscle disorders such as DMD [5]. In this latter pathology, due to the lack of the subsarcolemmal protein dystrophin, the continuous myofiber necrosis induces incessant myofiber regeneration, which gradually fails, while fat and fibrotic tissues increasingly infiltrate muscles. Such infiltrations contribute to muscle functional impairment and are considered as key limitations for cell and gene therapy.

In muscles, a subtype of mesenchymal stem cells called FAPs, for fibro-adipogenic progenitors, are thought to be the main progenitors of intramuscular myofibroblasts and adipocytes. This population of PDGFRα-positive mesenchymal progenitors, located in the perimysium among muscle fibers, was originally characterized as a major contributor to muscle homeostasis [6,7,8,9]. They are quiescent in normal muscles but proliferate upon muscle injury. FAPs do not generate myofibers, but support muscle regeneration through releasing several soluble factors such as Bmp3b [10], WISP-1 [11] and follistatin [12,13]. In a normal regeneration process, FAP activation at the site of damage is transient to prevent intramuscular accumulation of fibrotic and adipogenic tissues. However, in pathological conditions, such as during myopathies and DMD, the same muscle-resident FAPs proliferate and are maintained, resulting in muscular fibrosis [14,15]. This activity has been shown to be crucial for the muscle since depletion of FAPs in mice leads to muscle degeneration [16,17]. We, [18,19] and another group [9], established that the PDGFRα + CD56 − muscle progenitors can be considered as the human FAPs [20]. Our team highlighted a novel crosstalk between FAPs and myogenic progenitors (MPs) in humans that could be crucial in the formation of adipocyte and myofibroblast accumulation in dystrophic and aged skeletal muscle [19].

While FAP-mediated muscle fibrosis is widely studied in muscle diseases and homeostasis [21], the regulation of the fibrogenic potential of FAPs in humans is not well understood. Recent studies from us and others have identified several miRNAs recognized as critical effectors of the fibrogenic response to tissue injury by promoting TGFβ-induced lung fibroblast activation [22,23]. In the specific context of DMD, some of these “FibromiRs” have been implicated in the development of muscular fibrosis. For example, one of the most studied miRNA, miR-21, was associated with fibrosis in DMD [24]. Other FibromiRs, such as miR-199a-5p and miR-214-3p, both produced from the DNM3 Opposite Strand/Antisense (DNM3OS) non-coding RNA, have been shown to accelerate DMD [25], or to participate to the musculoskeletal metabolism [26], suggesting their central function in this disease. Thereby, such miRNAs should allow to define gene regulation networks implicated in the fibrogenic differentiation of FAPs and, therefore, in muscle fibrosis development.

The purpose of the present study is to investigate the role of FibromiRs in the fibrogenic potential of human FAPs to decipher molecular mechanisms of muscle fibrosis and to provide a basis for the development of new therapeutic strategies to prevent muscle fibrosis. First, we delineated the pool of miRNAs that are modulated during the fibrogenic differentiation of FAPs into myofibroblasts, by a global miRNA expression screening. This pool was compared with miRNAs deregulated in muscles from DMD patients with heterogeneous fibrotic infiltration. To further elucidate the collective pro- or anti-fibrotic role of the selected candidate miRNAs, silencing approaches in FAPs and analysis of their consequences for the fibrogenic process were performed. Specially, we identified miR-214-3p as a key regulator of TGFβ-induced FAP activation by targeting the FGFR pathway.

## 2. Materials and Methods

### 2.1. Reagents and Antibodies

Cell culture media, serum, buffer, and trypsin were purchased from Lonza (Verviers, Belgique) and cell culture reagents from Sigma-Aldrich Chimie (Saint-Quentin Fallavier, France).

The following monoclonal antibodies were used: mouse anti-β-Tubulin and mouse anti-α-SMA were purchased from Sigma-Aldrich (Saint Quentin Fallavier, France), rabbit anti-FGF Receptor 1 (9740) and rabbit anti-GSK3-β (12456) were from Cell Signaling (Ozyme, St Quentin en Yvelines, France). Anti-human PDGFRα (CD140a), anti-human CD56 antibodies, anti-β-Catenin (610153) and Fluorochrome-conjugated antibodies for flow cytometric analysis were purchased from BD Biosciences (Le Pont-de-Claix, France) (CD56-APC 345812; CD140a-PE 556002). Antibody against mouse coupled to Alexa Fluor 488 and antibody against rabbit coupled to Alexa Fluor 647 were from Life Technologies (Saint Aubin, France). DRAQ5 fluorescent probe (#62254) was purchased from Thermo Fisher Scientific and used at 20 µM. Human recombinant TGFβ1 (100-21) was purchased from PeproTech (Neuilly sur Seine, France). 

Specific FGFR1 inhibitor PD173074 was from STEMCELL Technologies (Grenoble, France). 

miRCURY LNA Knockdown probes: anti-miR-199a-3p (LNA-199a-3p), anti-miR-199a-5p (LNA-199a-5p), anti-miR-214-3p (LNA-214-3p and LNA-214-3p-FAM) and negative control anti-miR-159s LNA (LNA-CT) were ordered from QIAGEN. Lipofectamine RNAi MAX, Lipofectamine 2000 and miRNAs mimics (pre-miR-214-3p and control pre-miRNA) were purchased from Thermo Fisher Scientific (Illkirch, France).

### 2.2. Skeletal Muscle Samples and Progenitor Cell Amplification

Samples were obtained as res nullius from surgeries performed on healthy donors with the approval of the Centre Hospitalier Universitaire de Nice Review Board, according to the rules of the French Regulatory Health Authorities and with the informed consent of the parents for children and teenagers. DMD and age-matching control biopsies were obtained from Myobank-AFM Institut de Myology, Paris, France. Donor’s data are presented in Table 1, which includes the number of biopsies used in presented data. Progenitor cells were prepared by enzymatic digestion of muscle samples as previously described [18]. Cells were grown as adherent cells in Ham’s F10, 20% fetal bovine serum, 10 mM Hepes, 2.5 ng/mL basic fibroblast growth factor 2.1 µM dexamethasone, 100 U/mL penicillin, and 100 mg/mL streptomycin. Progenitors were sorted at passage 2 or 3 by flow cytometry as CD140a (PDGFRα)-positive cells and CD56-negative for FAPs, and CD56-positive and CD140a-negative cells for MPs. The purity of FAP and MP cells was checked via cytofluorometry with CD140a and CD56 antibodies, respectively (Figure S1). Cell separations were performed using a BD FACSAria II sorter with the BD FACSDiva software as previously described [18]. Cells were used until passage 10. For immunofluorescence analysis, cells were grown on glass cover slips.

A fibrotic score was attributed to each sample to aggregate the expression value of 3 fibrotic markers by qPCR following the formula, with *Exp Max* corresponding to the highest expression value in the cohort:

$$FS=1/3\left[\frac{Exp\left(COL1A1\right)}{Exp\;Max\left(COL1A1\right)}+\frac{Exp\left(FN1\right)}{Exp\;Max\left(FN1\right)}+\frac{Exp\left(ACTA2\right)}{Exp\;Max\left(ACTA2\right)}\right]$$

### 2.3. Growth and Differentiation of FAPs and MPs

The growth culture medium was Ham’s F10 medium supplemented with 20% FBS, 10 mM Hepes, 10^−6^ M dexamethasone, 2.5 ng/mL basic fibroblast growth factor, 100 U/mL penicillin, and 100 mg/mL streptomycin. The same differentiation medium (DM) was used for the differentiation of FAPs and MPs, consisting in Ham’s F10/F12/low-glucose DMEM with 2 mM glutamine (2v/1v/1v) supplemented with 1% horse serum (Gibco, Thermo Fisher Scientific, Villebon-sur-Yvette, France), 500 nM dexamethasone, 50 μM 1-methyl-3-isobutylmethyl xanthine (MIX), 7.5 μg/mL insulin, 7.5 μg/mL transferrin, 0.1 nM triiodothyronine, and 50 nM rosiglitazone (PPARγ agonist). Two days later, cells were placed in the same medium, lacking MIX and dexamethasone. Fibrogenic differentiation was obtained with the above DM complemented with 5 ng/mL TGFβ1. This differentiation medium was replaced every 2 days and cells were collected after 2 or 5 days of differentiation to analyze fibrogenesis.

### 2.4. Transfection Assays

FAP cells were grown in 20% FBS in Ham’s F10 medium and transfected at 60 to 70% confluency in 12-well plates using Lipofectamine RNAi MAX™ (Invitrogen) with miRCURY LNA Knockdown probes (QIAGEN) at a final concentration of 10 nM in Opti-MEM serum-free medium without antibiotic. After 24 h, transfection medium was removed and replaced by differentiation medium with 1.5 ng/mL TGFβ1 for 2 days in fibrogenic conditions. TGFβ1 concentration was decreased to 1.5 ng/mL for transfection experiments to reduce the basal level of TGFβ1 induction. Transfection efficiency was checked with fluorescent LNAs after quantification of transfected cells (Figure S2).

### 2.5. Immunofluorescence and Histological Staining

Cells were seeded on glass coverslips and treated as described in the text. For muscle biopsies from healthy and DMD donors, 5 µm-cryosections were performed. Cells and tissue sections were rinsed with PBS and fixed with ROTI Histofix 4% (Carl Roth, Lauterbourg, France) for 20 min at room temperature. Fixed cells and tissue sections were incubated in PBS with 3% bovine serum albumin (BSA) and 0.1% triton X-100 for 30 min at room temperature.

Cells and tissue sections were sequentially incubated with primary anti-αSMA, anti-PDGFRα or anti-CD56 antibodies overnight at 4 °C then with the corresponding secondary antibody for 45 min at room temperature. PBS wash was performed three times between all steps. Nuclei were stained with DRAQ5 and DAPI for tissue sections and cells, respectively. Cells were finally mounted in Mowiol and visualized with an Axiovert microscope (Carl Zeiss, Le Pecq, France) under oil immersion, and pictures were captured and treated with AxioVision software (Carl Zeiss).

### 2.6. Second Harmonic Generation (SHG) Imaging

After histological staining with anti-PDGFRα or anti-CD56 antibody and DRAQ5, imaging was performed on an LSM 780 NLO inverted Axio Observer.Z1 confocal microscope (Carl Zeiss Microscopy GmbH, Jena, Germany) using a Plan Apo 25X multi immersion (oil, glycerol, water) NA 0.8 objective. Fluorescence images were acquired using a laser 561 nm for Alexa 594 and a laser 640 nm for DRAQ5 (DNA). The SHG light source was a Mai Tai DeepSee (Newport Corp., Irvine, CA, USA) tuned at 880 nm. Forward SHG signal was detected with oil condenser (1.4 NA), bandpass filter 440/40 nm and transmission PMT. Backward SHG was collected on GaAsP (BIG) non-descanned module with 440/10 nm.

### 2.7. RNA Extraction and Reverse Transcription Quantitative Polymerase Chain Reaction

Total RNA was extracted using TRI Reagent (EUROMEDEX, Souffelweyersheim, France). The protocols of total RNA extraction, and quantitative RT-PCR of mRNAs were previously mentioned [18]. The housekeeping gene TATA box-binding protein (*TBP*) was used as reference. The 5′–3′sequences of forward and reverse primers were, respectively: CACGAACCACGGCACTGATT and TTTTCTTGCTGCCAGTCTGGAC for *TBP*, ACCTGCGTGTACCCCACTCA and CCGCCATACTCGAACTGGAA for *COL1A1*, CTGGCCGAAAATACATTGTAAA and CCACAGTCGGGTCAGGAG for *FN1*, TGCCTGCATGGGCAAGTGA and CTGGGCAGCGGAAACG for *ACTA2*, AGCGACCCTCACATCAAGCT and AGCACACACTCCTTTGATAGACACA for *FGF2*.

miRNAs expression was assessed using TaqMan MicroRNA Reverse Transcription Kit (Thermo Fisher Scientific) and TaqMan MicroRNA Assays (Thermo Fisher Scientific) as specified by the manufacturer. Real-time PCR was performed using Universal Master Mix II (Thermo Fisher Scientific). Expression levels of miR-199a-5p (assay ID 000498), miR-199a-3p (assay ID 002304), miR-214-3p (assay ID 002306), miR-29b (assay ID 00413) and let-7a (assay ID 002619) mature microRNAs were evaluated using comparative CT method. For normalization, RNU44 (assay ID 0010921) was used as endogenous control.

### 2.8. Small RNA Sequencing

Total RNAs were quantified using NanoDrop 1000 Spectrophotometer (Thermo Fisher Scientific, Waltham, MA, USA), and integrity of samples (RIN > 8) was evaluated using RNA nanochips on the Agilent 2100 Bioanalyzer Instrument (Agilent Technologies, Santa Clara, CA, USA). An amount of 0.5 µg of total RNA were ligated, reverse transcribed and amplified (13 cycles) with the reagents from the NEBNext Small RNA Library Prep Set for SOLiD. Amplified libraries were size-selected from 110 nt to 130 nt with the LabChip XT DNA 300 Assay Kit (Caliper Lifesciences). Libraries were subjected to 4 additional PCR rounds with the primers from the 5500 W Conversion Primers Kit (Life Technologies) and a second time the amplified libraries were size-selected from 140 nt to 160 nt with the LabChip XT DNA 300 Assay Kit (Caliper Life Sciences). Libraries were finally converted with the enzyme kit from the 5500 W Conversion Primers Kit (Life Technologies), quantified with the Bioanalyzer High Sensitivity DNA Kit (Agilent) and sequenced on SOLiD 5500XL (Life Technologies) with single-end 50b reads. All data were submitted to GEO repository under super series GSE157675 gathering GSE157668 series for small RNA sequencing of DMD samples and GSE157674 series for small RNA sequencing of FAP and MP differentiation.

### 2.9. Data Analysis

Raw reads were aligned to the human genome release hg19 with LifeScope v2.5.1 using the small RNA pipeline for miRNA libraries with default parameters. Quality check analyses were performed to assess reproducibility of data for the 2 series (Figures S3 and S4). Annotation files used for production of raw count tables correspond to miRBase v18. Differential expression analyses were performed using the DESeq package (Bioconductor) and statistical significance was assessed using exact tests. *p*-values were adjusted for multiple testing using the Benjamini and Hochberg method which controls the false discovery rate (FDR). Unsupervised hierarchical clustering was performed in R with the pheatmap package using Pearson′s correlation as distance and complete linkage.

### 2.10. Luciferase Assay

Molecular constructs were performed in psiCHECK-2 (Promega) by cloning annealed oligonucleotides derived from FGFR1 3′ UTR from base 1 to base 457 and from base 1650 to base 2100, upstream of the Renilla luciferase gene using the XhoI and NotI restriction sites. HEK293 cells were plated into 96-well plates and co-transfected using Lipofectamine 2000 (Thermo Fisher Scientific) with 0.2 µg of psiCHECK-2 plasmid constructs and pre-miR-214-3p or control pre-miRNA. After 48 h of transfection, Firefly and Renilla luciferase activities were measured using the Dual-Glo luciferase assay (Promega).

### 2.11. Immunoblot Analysis

Cells were lysed in RIPA buffer consisting of 50 mM Tris-HCl pH 8.0, 150 mM NaCl, 0.1% SDS, 0.5% sodium deoxycholate, 5 mM NaF, 2.5 mM Na_4_P_2_O_7_, 1% NP40, 2 mM sodium vanadate and protease inhibitor cocktail (Roche Diagnostics, Meylan, France). The protein content was determined according to the BCA method (Pierce BCA Protein Assay Kit, Thermo Fisher Scientific, Illkirch Graffenstaden, France #23227). Cell lysates were centrifuged at 13,000 *g* for 10 min at 4 °C and the supernatants were recovered. A total of 10 μg of proteins were resolved by 8% SDS-PAGE under reducing conditions and transferred to Immobilon-P polyvinylidene difluoride membrane (Merck Millipore, Fontenay sous Bois, France). The membranes were probed with the bound primary antibody that was detected by horseradish peroxidase-conjugated secondary antibody (Promega, Charbonnières-les-Bains, France) and visualized with Enhanced chemiluminescent detection kit Amersham (and a Bio-Rad ChemiDoc XRS+ imaging system (Bio-Rad, Marnes-la-Coquette, France)). The band intensity was measured using the Quantity One software (Bio-Rad) + imaging system. Full-length Western blots are provided in the supplementary data.

### 2.12. Statistical Analysis

Statistical analyses were performed using GraphPad Prism software. Results are given as mean ± SEM of at least three independent muscle biopsies in duplicate. Muscle biopsy samples were randomly chosen in our collection. Two-tailed unpaired Student’s t-test was used for single comparisons and one-way ANOVA, followed by Bonferroni post-hoc test which was used for multiple comparison. *p*-value < 0.05 was considered significant.

## 3. Results

### 3.1. Small RNA Seq of DMD Muscle Biopsies Identifies an miRNA Signature Associated with Fibrotic Status

**Figure 1:**
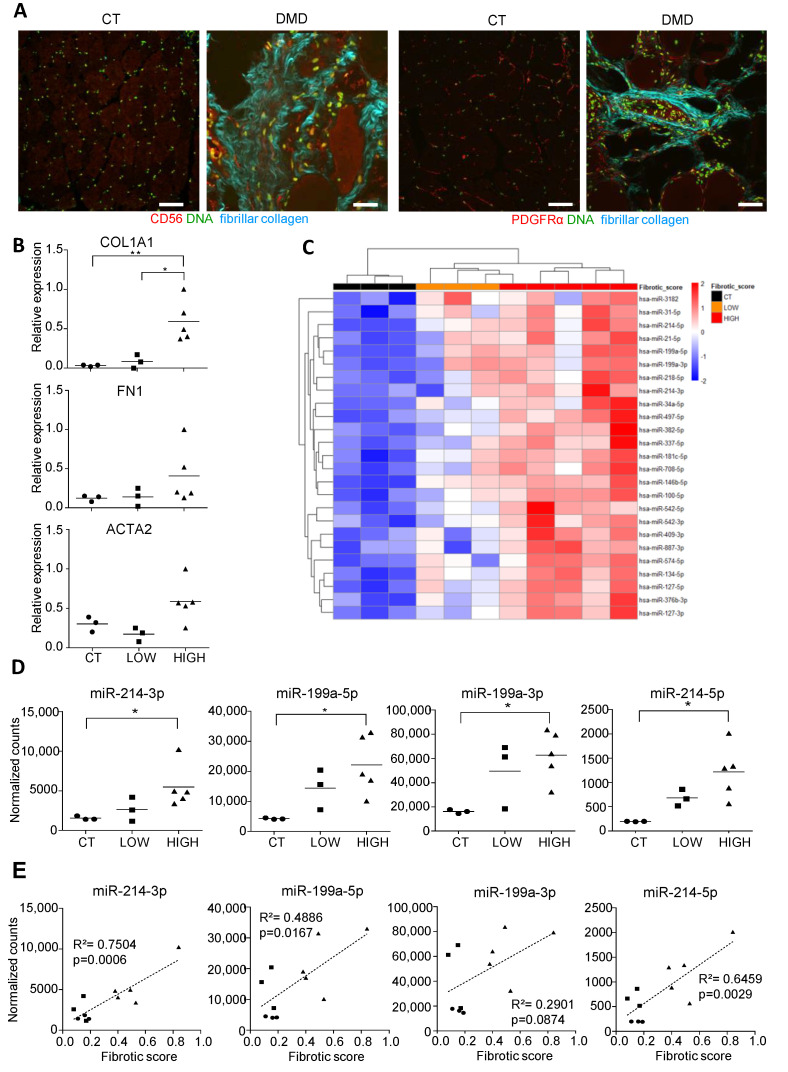
miRNA expression in DMD versus control muscle biopsies. (**A**) Intramuscular fibrillar collagen was visualized in cyan by second-harmonic generation imaging on cryostat sections from one healthy (CT) or DMD donor. Sections were co-stained with CD56 or PDGFRα (red) and DRAQ5 (green). Representative images of staining in healthy or DMD donors are shown. (**B**) Relative expression of *ACTA2*, *COL1A1* and *FN1* in DMD (A to I) and 3 healthy (CT1 to CT3) muscle samples sorted by fibrotic score. * *p* < 0.05, ** *p* < 0.01. (**C**) Hierarchical clustering of 8 DMD muscle biopsies and 3 age-matching control muscle biopsies using a subset of 17 miRNAs enables to separate samples according to their fibrotic status. Heat map representing the normalized log2 reads number scaled using a Z-score for the best 25 miRNAs whose expression correlated with a fibrotic score (r > 0.5). Mature miRNAs from miR-199a~214 cluster was highlighted in red. (**D**) Expression level of DNM3OS associated miRNAs miR-214-3p, -199a-5p, -214-3p and -199a-3p in each DMD muscle biopsies and 3 age-matching control muscle biopsies sorted by fibrotic score. Data are expressed as normalized count. * *p* < 0.05. (**E**) Pearson’s correlation scatter plot of DNM3OS-associated miRNAs miR-214-3p, -199a-5p, -214-3p and -199a-3p expression and fibrotic score for each 8 DMD muscle biopsies and 3 age-matching control.

Eight DMD muscle biopsies and three age-matching control muscle biopsies (Table 1) were selected on the basis of their fibrosis status. Fibrosis was evaluated on cryostat sections by (i) second harmonic generation, a method dedicated to visualizing the highly organized collagen (Figure 1A and Figure S5) and (ii) expression of fibrosis markers *ACTA2*, *COL1A1* and *FN1* by RT-qPCR (Figure 1B). We defined a fibrotic score based on the *ACTA2*, *COL1A1* and *FN1*-normalized expression (from 0 to 1) and the DMD samples were divided into two subsets named “Low” (*n* = 3, with a fibrotic score equivalent to that of control biopsies and <0.25) and “High” (*n* = 5, with a fibrotic score >0.25). RNA samples were extracted from biopsies and subjected to small-RNA-seq. 

The data shown in Figure 1C and Table S1 indicate that the miRNome content of DMD biopsies clearly differs from control muscles. Unsupervised hierarchical clustering of the samples using this miRNA subset selectively expressed in DMD biopsies separated high fibrotic from low fibrotic biopsies. Among these deregulated miRNAs, a subset corresponded to several “FibromiRs”, including the four mature miRNAs produced by the DNM3OS transcript on chromosome 1 (cluster miR-199a~214) (Figure 1D). Among the four mature miRNAs produced by the DNM3OS included in the signature, miR-214-3p had the best Pearson’s correlation value with the fibrotic score (Figure 1E).

### 3.2. FAPs and MPs Display a Distinct miRNA Expression Profile That is Modulated in Response to TGFβ1

**Figure 2:**
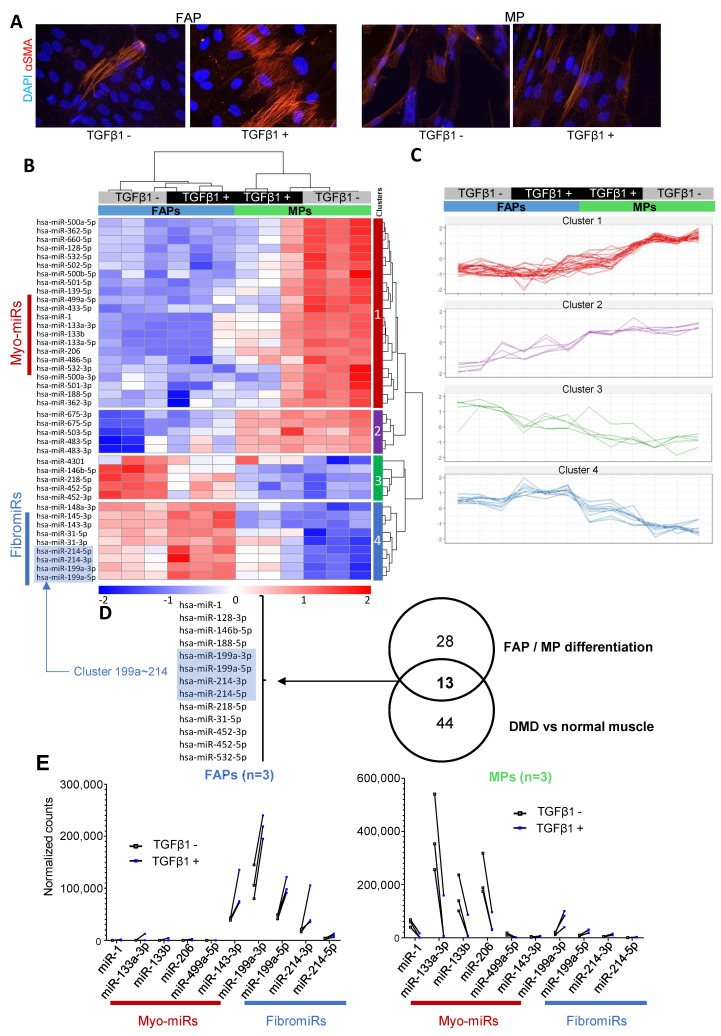
miRNA expression in control and TGFβ1-treated FAPs and MPs. **(A)** α-SMA immuno-labelling revealed α-SMA-expressing fibers (red) present in FAPs treated with TGFβ1 (5 ng/mL) but not in MPs. Nuclei were labelled with DAPI (blue). The images were representative for all fields. (**B, C**) Hierarchical clustering of control and TGFβ1-stimulated FAPs and MPs samples using the most modulated miRNAs differentially expressed between control or TGFβ1-treated FAPs and MPs (*n* = 3). (**B**) Heat map representing the normalized log2 reads number scaled using a Z-score and (**C**) expression profiles for the main 4 clusters. (**D**) Venn diagram indicates the intersection of miRNAs modulated between DMD and healthy muscle with miRNAs modulated during TGFβ1-induced FAP/MP differentiation. Mature miRNAs from miR-199a~214 cluster is highlighted in blue. (**E**) Typical profiles of a subset of miRNA significantly deregulated in control and TGFβ1-treated FAPs and MPs as measured by small RNA sequencing. Values represent the normalized reads number in each condition (*n* = 3).

Since FAPs are recognized as one of the main sources of myofibroblasts in skeletal muscles [27], we assessed whether such subsets of miRNAs were differentially expressed in FAPs compared to myogenic progenitors (MPs) or deregulated during fibrotic differentiation. FAPs and MPs from three healthy skeletal muscles were subjected—or not—to TGFβ1 for 48 h. An immunofluorescent analysis of αSMA stress fibers revealed fibrogenic phenotypes of FAPs, but not MPs, upon TGFβ1 exposure (Figure 2A).The expression of several pro- or anti-fibrotic miRNAs was measured using qRT-PCR. Data indicated that TGFβ1 induced upregulation of pro-fibrotic miRNAs in FAPs, especially DNM3OS-associated miRNAs miR-214-3p and miR-199a-5p, while anti-fibrotic miRNAs, Let-7a and miR-29b, were only induced in MPs (Figure S6).

From these three muscle biopsies, the small RNA-seq of FAPs and MPs treated—or not—by TGFβ1, confirmed that FAPs and MPs expressed very different subsets of miRNAs. A subset of 59 miRNAs accurately discriminated FAP- and MP-derived samples (Table S2). Moreover, additional miRNAs were differentially regulated by TGFβ1, pointing out a specific miRNome modulation in the two muscle progenitors during differentiation (Figure 2B,C). Figure 2D illustrates some of these typical miRNA profiles that could be grouped into four main subsets. Expression of several FibromiRs, including the cluster miR-199a~214, was significantly higher in FAPs compared to MPs and was strongly upregulated by TGFβ1 (Figure 2E). Conversely, several miRNAs, highly enriched in cardiac and/or skeletal muscle [28] (referred to as myomiRs), including miR-1, miR-133a/b, miR-206, and miR-499a, were specifically found expressed by MPs (Figure 2E). Overall our data underlined the specificity of expression of miRNAs in FAPs and MPs as well as the strong influence of TGFβ1 on the expression of a large subset of these miRNAs in the two cell progenitors.

### 3.3. Knockdown of miR-214-3p Inhibits FAP Fibrogenesis

Since the expression of the mature miRNAs transcribed from the cluster miR-199a~214 was strongly upregulated by TGFβ1 in FAPs (Figure 2C) and also altered in DMD (Figure 1C), we tested the effect of LNA-antisense oligonucleotides (ASOs) directed against the three most-expressed mature miRNAs of the cluster (LNA-199a-3p, LNA-199a-5p or LNA-214-3p) on FAP differentiation.

**Figure 3:**
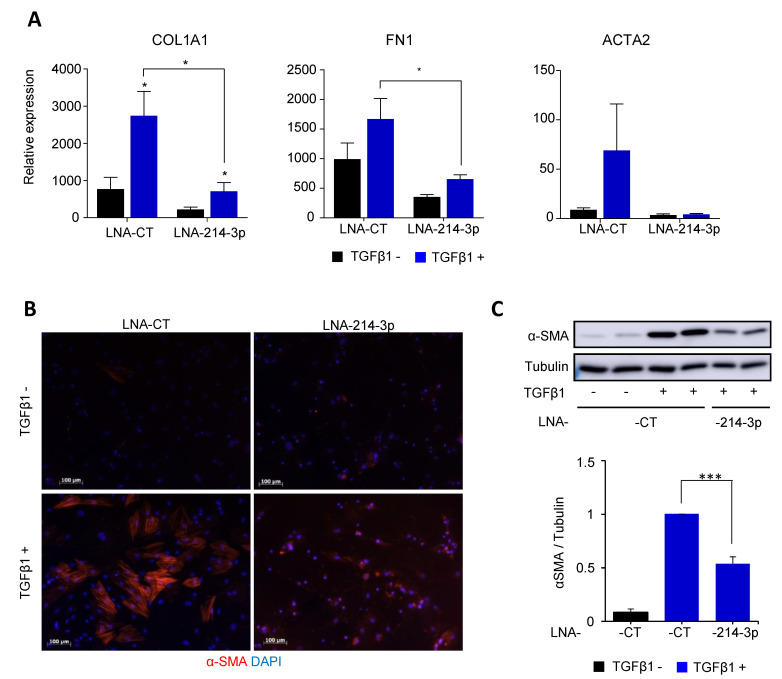
Knockdown of miR-214-3p inhibits FAP fibrogenesis. FAPs were transfected with 10 µM LNA-214-3p or LNA-CT for 24 h, then cultured with differentiation medium supplemented with 1.5 ng/mL TGFβ1 for 2 days. (**A**) *COL1A1*, *FN1* and *ACTA2* fibrogenic genes were measured by quantitative RT-PCR versus *TBP* housekeeping gene. Experiments was performed on three distinct biopsies. Data are express as mean ± SEM. * *p* < 0.05. (**B**) α-SMA immuno-labelling revealed α-SMA-expressing fibers (red) present in myofibroblasts. Nuclei were labelled with DAPI (blue). The images are representative for all fields. The white bar represents 100 μm. (**C**) α-SMA proteins were detected by immunoblot. Tubulin was used as loading control. One of three independent replicates is shown. Quantification is shown as mean ± SEM. *** *p* < 0.001.

Transient transfection with fibromiR inhibitors was performed in the presence or absence of TGFβ1. While LNA-199a-3p or LNA-199a-5p did not show reduction in *COL1A1*, *FN1* and *ACTA2* expression profiles compared to a control inhibitor (LNA- CT), LNA-214-3p strongly affected the three fibrotic markers (Figure S7). Knockdown of miR- 214-3p was repeated in FAP cells cultured in TGFβ1-induced fibrogenic conditions (Figure 3). 

As expected, TGFβ1 stimulated the expression of fibrotic markers with an increase of 2-, 3- and 10-fold in *FN1*, *COL1A1* and *ACTA2*, respectively, in LNA-CT transfected FAPs. In TFGβ1-supplemented conditions, LNA-214-3p significantly affected *COL1A1*, *FN1* and *ACTA2* gene expression (Figure 3A) with an inhibition of 3.8-, 2.5- and 17-fold compared to LNA-CT, respectively. Of note, LNA-miR-214-3p also affected basal expression of fibrotic markers in FAPs (Figure 3A). Finally, after 48 h of fibrogenic differentiation in the presence of TGFβ1, LNA-214-3p treatment strongly inhibited the formation of myofibroblasts, as shown by the decreased expression of the α-SMA protein detected by immunostaining (Figure 3B) and immunoblot (Figure 3C). In brief, knockdown of miR-214-3p inhibited TGF-β-induced FAP fibrogenesis.

### 3.4. MiR-214-3p Mediates TGFβ1-Induced FAP Activation by Targeting the FGFR1 Pathway

To investigate the mechanism of action of miR-214-3p, we first analyzed the β-catenin pathway, as we recently showed that miR-214-3p promoted this SMAD-independent profibrotic component of TGF-β signaling by targeting GSK-3β in TGFβ1-stimulated pulmonary fibroblasts [23]. However, while treatment of FAPs with LNA-214-3p increased expression levels of GSK3-β (Figure S8A), no significant effect could be measured on β-catenin activation since TGF-β did not significantly induce β-catenin nuclear translocation in our experimental condition of FAP fibrogenic differentiation (Figure S8B).

**Figure 4:**
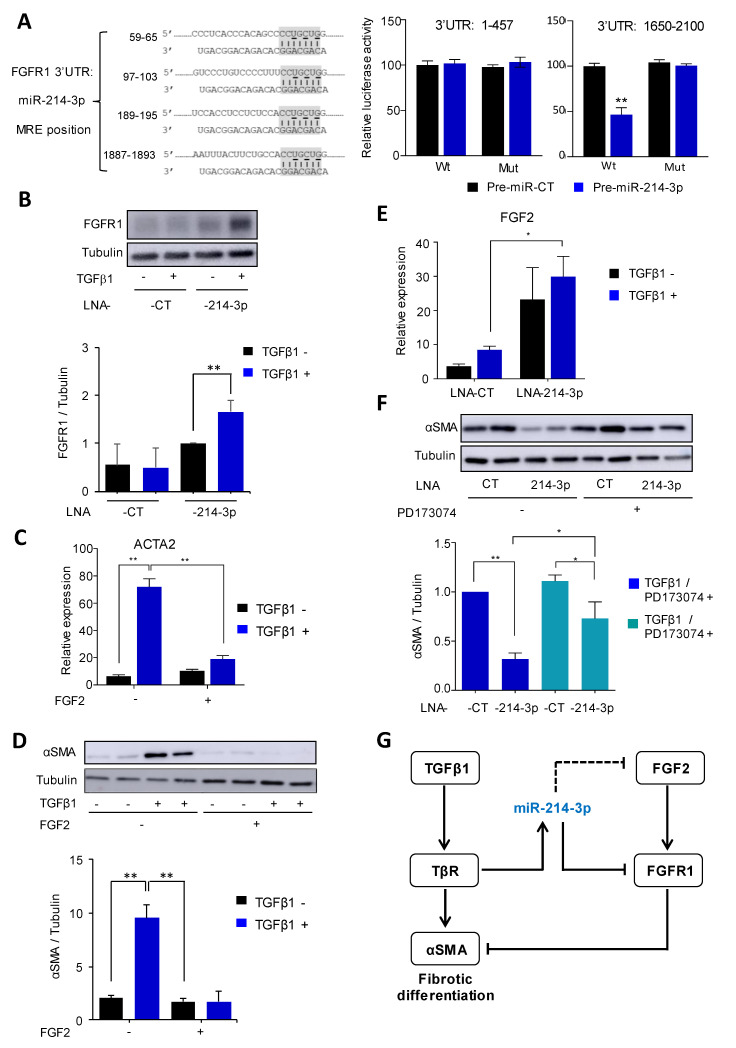
miR-214-3p mediates TGFβ1-induced FAP activation by targeting FGFR pathway. (**A**) Position of 4 miR-214-3p putative target site on FGFR1 3′UTR from TargetScan (19). The seed region of miR-214-3p is highlighted in grey and complementarities are indicated. Bases that were mutated in psiCHECK-2 constructs (MUT) are underlined. Pre-miR-214-3p or control Pre-miR-CT and hFGFR1 3′UTR (from base 1 to base 457 and from base 1650 to base 2100)-derived psiCHECK-2 constructs were transfected in HEK293 cells. All Renilla luciferase activities were normalized with firefly luciferase activity. (*n* = 3). ** *p* < 0.01. (**B**) FGFR1 protein was detected by immunoblot in FAPs transfected with LNA CT or LNA-214-3p and then stimulated—or not—with TGFβ1 for 2 days. Tubulin was used as loading control. One of three independent replicates is shown. Quantification is shown as mean ± SEM. ** *p* < 0.01. (**C**) ACTA2 mRNA expression was measured by quantitative RT-PCR in FAPs co-stimulated by TGFβ1 (5 ng/mL) and FGF2 (10 ng/mL)—or not. *TBP* was used as housekeeping gene. Experiments were performed on three distinct biopsies. Data are expressed as mean ± SEM. ** *p* < 0.01. (**D**) αSMA was detected by immunoblot in FAPs stimulated with the FGFR inhibitor PD173074 at 75 nM for 10 min and then transfected with LNA-CT or LNA-214-3p for 24 h before the differentiation phase in fibrogenic condition induced by 1.5 ng/mL of TGFβ1 for 2 days. Tubulin was used as loading control. One of three independent replicates is shown. Quantification is shown as mean ± SEM. ** *p* < 0.01. (**E**) *FGF2* mRNA expression was measured by quantitative RT-PCR in FAPs, treated with LNA-CT or -214-3p (10 µM) and then stimulated—or not—with 1.5 ng/mL of TGFβ1 for 2 days. *TBP* was used as housekeeping gene. Experiments were performed on three distinct biopsies. Data are expressed as mean ± SEM. * *p* < 0.05. (**F**) αSMA was detected by immunoblot in FAPs stimulated with the FGFR inhibitor PD173074 at 75 nM for 10 min and then transfected with LNA-CT or LNA-214-3p for 24 h before the differentiation phase in fibrogenic condition induced by 1.5 ng/mL of TGFβ1 for 2 days. Tubulin was used as loading control. One of three independent replicates is shown. Quantification are shown as mean ± SEM. ** *p* < 0.01, * *p* < 0.05. (**G**) General representation of the mechanism of action of miR-214-3p in FAPs. The induction of miR-214-3p by TGFβ1 mediates the inhibition of the anti-fibrotic FGF2/FGFR1 signaling through the direct targeting of FGFR1 by miR-214-3p.

By looking at the potential molecular mechanisms that could explain the inhibitory effect of miR-214-3p silencing on FAP differentiation, we focused on FGF signaling. Indeed, FGF-2 is a well described inhibitor of myofibroblast differentiation [29], and its receptor FGFR1 was described as a potential target of miR-214-3p. FGFR1 has four predicted binding sites for miR-214-3p in its 3′UTR according to TargetScan [30] (Figure 4A). Consistent with previous studies [28,30], we validated the position 1887–1893 as a functional interaction site (Figure 4A). Our results confirm this interaction and the regulation of FGFR1 expression by miR-214-3p as the FGFR1 protein level was strongly increased after LNA-miR-214-3p transfection compared to control (Figure 4B).

We next investigated the potential inhibitory action of miR-214-3p on the antifibrotic FGF2 pathway. As expected, FGF2 prevented FAP fibrogenic differentiation as evidenced by the strong reduction in TGF-β-mediated α-SMA induction (Figure 4C,D). *FGF2* mRNA expression was then measured in FAPs cultured in fibrogenic conditions for 48 h. *FGF2* mRNA was overexpressed 3.5-fold when FAPs were treated with LNA-214-3p compared to LNA-CT (Figure 4E). This result suggested that the FGF2 autocrine signaling pathway may be targeted by miR-214-3p. Since the *FGF2* transcript does not contain any predicted miR-214-3p response elements, it appears likely that the effect of miR-214-3p silencing is indirect.

To investigate the impact of FGFR1 as an miR-214-3p target on FAP differentiation, cells were submitted to FGFR inhibition prior to transfection. PD173074 is a well characterized FGFR1 inhibitor [31] and was tested in fibrogenic conditions (with TGFβ1) in the presence of the LNA-214-3p inhibitor or an LNA-CT (Figure 4F). No effect of PD173074 could be observed on α-SMA protein expression when cells were treated with LNA-CT. 

However, the inhibitory effect of LNA-214-3p on α-SMA protein expression was significantly alleviated in the presence of the PD173074 inhibitor with a decrease in the α-SMA expression of 1.5-fold (Figure 4F). In summary, the FGFR inhibitor PD173074 partially rescued miR-214-3p knockdown-mediated α-SMA inhibition, indicating that FGFR1 is a functional target of miR-214-3p, preventing the signaling of the anti-fibrotic FGF2 pathway during FAP fibrogenic differentiation (Figure 4G).

## 4. Discussion

ECM synthesis has a major role in muscle repair following injury [32]. Indeed, the transient ECM deposition in damaged muscle is required to complete MP myogenesis and acts as a scaffold for regenerating myofibers. Fibrosis is an excessive deposition of ECM that is a feature of skeletal muscles in patients with DMD. The structure of the muscles is disorganized, resulting in muscular weakness dramatically reducing life expectancy of the patients and limiting the success of therapies. Muscle fibrosis is the result of an increase in differentiation of myofibroblasts, which is being regulated by various factors. In the last decade, miRNAs emerged as important regulators of fibrosis in several tissues, called fibromiRs [33], and of skeletal muscle development and homeostasis, named myomiRs [34].

Here, we first determined the pattern of miRNA expression in muscles biopsies obtained from healthy or DMD donors. As previously described [35], miRNAs found in biopsies from DMD patients differ from those found in biopsies from healthy donors. In particular, well-established myomiRs such as miR-1 and miR-486 are downregulated in DMD patients, while fibromiRs, such as miR-214, miR-199a and miR-31 are overexpressed in DMD muscle biopsies. Our results are consistent with those obtained in muscles of *mdx* mice that are widely used as the animal model of DMD pathology [25,36], as well as in human muscle biopsies [25,35]. The originality of our study is the link between miRNA expression and the fibrotic status of DMD muscles. Indeed, a fibrotic index, based on the normalized expression of key fibrotic genes (COL1A1, FN1 and ACTA2), was established to classify the eight DMD biopsies used. This index revealed a significant heterogeneity in the extent of fibrosis in DMD muscles from different patients and a subset of 25 miRNAs enabled to separate the biopsies according to their fibrotic status (Figure 1C). Among these deregulated miRNAs, a subset corresponded to several “FibromiRs”, including the four mature miRNAs produced by the DNM3OS transcript on chromosome 1 (cluster miR-199a~214) (Figure 1D). Of note, miR-214-3p had the best Pearson’s correlation value with the fibrotic score, suggesting a key function of this miRNA in muscle fibrogenesis (Figure 1E).

FAPs reside in uninjured skeletal muscles in a quiescent state and become activated following injury [7,8]. They play crucial roles in muscle repair promoting MP differentiation into myofibers and producing the transitory ECM. To avoid excessive deposition of adipocytes and fibrosis in regenerating muscle, the number of FAPs declines mainly by activation of apoptosis. However, FAPs can persist in high numbers leading to fibrotic degeneration of muscle such as in DMD [9,37]. As FAPs are one of the main cells responsible for muscle fibrosis and display a fibrotic potential, we investigated (i) the pattern of miRNA expression in healthy FAPs and (ii) documented the evolution of the miRnome in response to TGFβ1. In contrast to FAPs, MPs are unable to differentiate into myofibroblasts after TGFβ1 stimulation. Consequently, the miRNA expression patterns in FAPs were compared to those in MPs. The hierarchical clustering of control and TGFβ1-stimulated FAPs and MPs using the most modulated miRNAs quantified by small RNA-seq highlighted the existence of four miRNA clusters (Figure 2B). MiRNAs present in clusters one and two were essentially expressed in MPs and absent in FAPs in control conditions. Upon TGFβ1 stimulations, miRNAs of the cluster one were downregulated and those of the cluster two were largely unaffected. The miRNAs of cluster one were well-established muscle-specific myomiRs, involved in the skeletal muscle proliferation, differentiation and regeneration [38]. Regulation of these myomiRs is controlled by key myogenic regulatory factors, including myogenic differentiation 1 (MyoD) and myogenin, as well as myocyte enhancer factor 2 (MEF2). Our data confirmed that they are strongly inhibited by TGFβ1 and are consistent with the antagonistic role of TGFβ1 and myomiRs in muscle homeostasis, already identified in pathological states [39], 10-days resting [40] or exercising–healthy subjects [41]. MiRNAs present in clusters three and four were expressed in FAPs and mostly absent in MPs in control conditions. Upon TGFβ1 stimulation, the levels of miRNAs of the cluster three were downregulated, while those of cluster four were up-regulated. The miRNAs of cluster four were referred to as fibromiRs [33]. This class of miRNAs is aberrantly expressed in fibrotic tissues and their expression is positively regulated by TGFβ1.

In cluster 4, the DNM3OS-derived miRNAs (miR-214-5p/3p, miR199-5p/3p) were strongly upregulated by TGFβ1 in FAPs. We, therefore, decided to focus on this subset of miRNA for functional studies. The utilization of ASOs directed against the three most-expressed mature miRNAs of the cluster (LNA-199a-3p, LNA-199a-5p or LNA-214-3p) showed that the LNA-214-3p was the most potent inhibitor of FAP differentiation. The knockdown of miR-214-3p was repeated in FAP cells cultured in TGFβ1-induced fibrogenic conditions and showed a marked inhibition of expression of COL1A1, FN1 and ACTA2, as well as FAP differentiation. These results suggest a strong involvement of miR-214-3p in the process of FAP fibrogenesis.

Amongst its many other predicted targets, miR-214-3p has been shown to be a direct target of FGFR1, to reduce FGFR1 protein levels and to inhibit the FGF2/FGFR1 signaling pathway [42,43]. This interaction has been shown to reduce osteogenic differentiation [42] and to decrease proliferation, migration and invasion of non-small-cell lung cancer cells [43]. Considering the well-established anti-fibrotic and pro-myogenic actions of the FGF2/FGFR1 signaling pathway [44,45], we examined whether miR-214-3p-induced FGFR1 inhibition could play a role in the profibrotic action of miR-214-3p. Our results suggest that the FGF2/FGFR1 axis is involved in a retro inhibitory loop in which autocrine/paracrine secretion of FGF2 activates FGFR1, leading to the inhibition of the TGFβ/TGFβ receptor profibrotic effect. In this context, miR-214-3p blocks this antifibrotic loop by inhibiting both FGF2 and FGFR1 expression (Figure 4G). Depending on the context, miR-214-3p, thus, appears as a repressor of the TGFβ pathway by acting on either negative (FGF2/FGFR1) or positive (Wnt/β-catenin) [23] regulatory pathways. Indeed, Wnt5a expression is downregulated in mdx mice and the WNT5a/GSK3/β-catenin axis inhibits adipocyte differentiation of FAPs [46]. While our experiments showed that miR-214-3p-dependent regulation of FAP differentiation did not involve the translocation of β-catenin into the nucleus, it will be interesting to investigate the role of the Wnt/β-catenin pathway in FAP fibrogenesis independently to miR-214-3p. 

The lack of expression of myomiRs in FAPs has also been documented and histone deacetylase inhibitors have been proposed as a potential therapeutic strategy through their ability to derepress a latent myogenic program in FAPs from DMD muscles at early stages of the disease [47]. The present study offers the possibility to envisage new therapeutic strategies based on the inhibition of fibromiRs. Such strategies would exploit the extensive development in the chemistry of ASOs [48]. In this strategy, modified nucleic acids can penetrate cells and modulate gene expression through different mechanisms based either on target site occupancy or on enzymatic RNA degradation. Many ASOs are currently in clinical trials and even commercialized in a wide range of complex disorders, including Crohn’s disease and cancer, as well as inherited diseases such as DMD [49]. In the light of our results, we advocate that ASOs targeting of the DNM3OS non-coding RNA and/or of the mature miRNAs produced from this lncRNA (particularly miR-214-3p) represents an attractive new therapeutic approach to treat muscle fibrosis. Indeed, it could be envisioned that mir214-3p targeting may have at least two beneficial effects on muscles of DMD patients, first decreasing fibrosis by inhibiting the differentiation of FAPs into myofibroblasts and second, promoting myogenesis by increasing the production of FGF-2. Further analyses will be necessary to assess this hypothesis.

## Figures and Tables

**Table 1:** Characteristics of healthy and DMD patients.

Name	Gender	Age	Muscle Origin	Use
**DMD donors**				
A	M	14	paravertebral	Figure 1
B	M	12	paravertebral	Figure 1
C	M	13	paravertebral	Figure 1
D	M	15	paravertebral	Figure 1
E	M	15	paravertebral	Figure 1
F	M	14	paravertebral	Figure 1
G	M	16	paravertebral	Figure 1
H	M	15	paravertebral	Figure 1
**Healthy Donors**				
CT1	M	14	intervertebral	Figure 1
CT2	M	<10		Figure 1
CT3	M	<10		Figure 1
1008	F	43		Figure 2
1019	M	30		Figure 2
1169	F	32		Figure 2
D5	M	17	gluteus maximus	Figure 3
D9	M	57		Figure 3
K12-2	M	<10		Figure 4
K14-1	M	3	inguinal	Figure 4
K13-6	M	4	gluteus maximus	Figure 4

## Data Availability

All data were submitted to the GEO repository under super series GSE157675 gathering GSE157668 series for small RNA sequencing of DMD samples and GSE157674 series for small RNA sequencing of FAP and MP differentiation.

## Supplementary Materials

The following are available online at https://www.mdpi.com/article/10.3390/cells10071832/s1, Table S1: list of differentially expressed miRNAs in DMD versus healthy muscle biopsies, Table S2: list of differentially expressed miRNAs in FAPs versus MPs, Figure S1: immunophenotype controls showing the purity of FAPs and MPs populations performed by flow cytometry, Figure S2: visualization and quantification of fluorescent LNA-214-4p-FAM in FAPs, Figure S3: overview of miRNAs data expression in DMD versus control muscle biopsies, Figure S4: overview of miRNA expression data in control and TGFβ1-treated FAPs and MPs, Figure S5: collagen, CD56 and PDGFRα visualization in DMD and healthy control samples, Figure S6: pro-fibrotic miR-21 -199a-5p -214-3p and anti-fibrotic Let-7a and miR-29b expression and fold induction in MPs and FAPs stimulated—or not—with TGFβ1 (5 ng/mL) for 48 h, Figure S7: effect of miR-199a-3p, miR-199a-5p and miR-214-3p knockdown on FAP differentiation, Figure S8: impact of miR-214-3p on GSK3-β/β-Catenin axis.
