# Supplementary material for: The FibromiR miR-214-3p Is Upregulated in Duchenne Muscular Dystrophy and Promotes Differentiation of Human Fibro-Adipogenic Muscle Progenitors
Source: Cells. 2021 Jul 20;10(7):1832. doi: 10.3390/cells10071832 (PMC8303294; doi:10.3390/cells10071832)
Supplement: Supplementary file 1 [file cells-10-01832-s001.zip › cells-1264410-supplementary.pdf]

Supplementary Materials

The FibromiR miR-214-3p is upregulated in Duchenne Muscular Dystrophy and promotes differentiation of human fibro-adipogenic muscle progenitors

Nicole Arrighi, Claudine Moratal, Grégoire Savary, Julien Fassy, Nicolas Nottet, Nicolas Pons, Noémie Clément, Sandy Fellah, Romain Larrue, Virginie Magnone, Kevin Lebrigand, Nicolas Pottier, Claude Dechesne, Georges Vassaux, Christian Dani, Pascal Peraldi and Bernard Mari

Supplementary Table 1. List of differentially expressed miRNAs in DMD *versus* healthy muscle biopsies. The table indicates the MIMAT mature entry for each sequence, the Log2 Base Mean, the Log2 Ratio between DMD and control muscles biopsies, the adjusted p.value and the correlation with the fibrotic score. Thresholds used for selection of the miRNA candidates are log2 Base Mean>5, Log2 FC>1 and adj.Pval <0.05.

Name	description	log BaseMean	log2 FC DMD vs CT	pVal.Adj DMD vs CT	Pearson Fibrotic score
hsa-let-7d-3p	MIMAT0004484	9.69	-1.25	4.90E-02	-0.21
hsa-miR-1	MIMAT0000416	16.93	-1.99	8.37E-03	-0.36
hsa-miR-100-5p	MIMAT0000098	12.60	1.69	1.04E-03	0.64
hsa-miR-127-3p	MIMAT0000446	10.43	1.94	2.48E-03	0.61
hsa-miR-127-5p	MIMAT0004604	7.93	2.36	5.49E-05	0.54
hsa-miR-128-3p	MIMAT0000424	11.77	-1.41	4.48E-02	-0.03
hsa-miR-134-5p	MIMAT0000447	7.77	2.27	4.15E-05	0.55
hsa-miR-146a-5p	MIMAT0000449	9.71	1.69	2.14E-02	0.21
hsa-miR-146b-5p	MIMAT0002809	12.06	3.95	3.76E-13	0.54
hsa-miR-16-1-3p	MIMAT0004489	6.83	2.06	2.29E-03	0.19
hsa-miR-181c-5p	MIMAT0000258	7.65	1.59	4.53E-04	0.62
hsa-miR-188-5p	MIMAT0000457	8.82	1.32	4.77E-02	0.48
hsa-miR-193a-3p	MIMAT0000459	9.08	1.40	1.71E-03	0.48
hsa-miR-199a-3p	MIMAT0004563	15.50	1.71	1.71E-03	0.57
hsa-miR-199a-5p	MIMAT0000231	13.89	2.02	2.26E-04	0.68
hsa-miR-214-3p	MIMAT0000271	11.83	1.36	4.99E-02	0.82
hsa-miR-214-5p	MIMAT0004564	9.63	2.21	2.88E-05	0.76
hsa-miR-21-5p	MIMAT0000076	16.86	2.31	2.98E-04	0.62
hsa-miR-218-5p	MIMAT0000275	9.28	1.30	2.14E-02	0.75
hsa-miR-221-3p	MIMAT0000278	11.82	1.22	1.72E-02	0.26
hsa-miR-22-5p	MIMAT0004495	12.59	-1.80	4.03E-03	-0.19
hsa-miR-30a-3p	MIMAT0000088	7.44	-1.61	9.43E-03	-0.35
hsa-miR-30b-5p	MIMAT0000420	14.15	-1.35	3.19E-02	-0.29
hsa-miR-30c-5p	MIMAT0000244	14.24	-1.51	2.54E-02	-0.26
hsa-miR-30e-3p	MIMAT0000693	10.36	-1.59	1.51E-02	-0.37
hsa-miR-31-5p	MIMAT0000089	6.82	3.14	2.60E-05	0.73
hsa-miR-3182	MIMAT0015062	12.82	3.24	2.48E-05	0.51
hsa-miR-320a	MIMAT0000510	13.44	1.28	1.74E-02	0.21
hsa-miR-320b	MIMAT0005792	9.48	1.57	1.33E-02	0.20
hsa-miR-320c	MIMAT0005793	9.06	1.42	1.86E-02	0.18
hsa-miR-337-5p	MIMAT0004695	9.86	1.53	1.65E-02	0.57
hsa-miR-34a-5p	MIMAT0000255	10.13	1.75	7.00E-03	0.79
hsa-miR-365b-3p	MIMAT0022834	13.14	-1.90	2.61E-03	-0.14
hsa-miR-376a-3p	MIMAT0000729	8.04	1.28	2.47E-05	0.48

hsa-miR-376b-3p	MIMAT0002172	7.99	1.88	2.36E-03	0.58
hsa-miR-376c-3p	MIMAT0000720	11.42	1.31	1.24E-02	0.48
hsa-miR-378a-5p	MIMAT0000731	13.30	-1.37	1.72E-02	-0.34
hsa-miR-381-3p	MIMAT0000736	6.81	1.29	2.47E-02	0.46
hsa-miR-382-5p	MIMAT0000737	7.24	1.69	1.72E-02	0.61
hsa-miR-409-3p	MIMAT0001639	7.74	1.59	2.24E-02	0.62
hsa-miR-410-3p	MIMAT0002171	7.42	1.87	1.72E-02	0.50
hsa-miR-452-3p	MIMAT0001636	7.85	1.86	4.03E-03	0.41
hsa-miR-452-5p	MIMAT0001635	10.02	2.16	2.60E-05	0.35
hsa-miR-486-3p	MIMAT0004762	8.56	-1.63	3.22E-02	-0.35
hsa-miR-497-5p	MIMAT0002820	8.96	1.11	4.11E-02	0.72
hsa-miR-5095	MIMAT0020600	7.22	1.34	2.29E-03	0.17
hsa-miR-532-5p	MIMAT0002888	10.44	1.29	1.51E-02	0.33
hsa-miR-542-3p	MIMAT0003389	7.77	1.97	6.02E-03	0.64
hsa-miR-542-5p	MIMAT0003340	7.25	2.56	8.23E-04	0.61
hsa-miR-551b-3p	MIMAT0003233	7.79	2.05	5.81E-03	0.29
hsa-miR-574-5p	MIMAT0004795	8.54	1.00	4.13E-02	0.65
hsa-miR-664a-3p	MIMAT0005949	8.05	-1.80	2.67E-02	0.01
hsa-miR-671-5p	MIMAT0003880	7.95	2.22	3.68E-04	0.29
hsa-miR-708-5p	MIMAT0004926	7.40	1.80	2.47E-03	0.66
hsa-miR-885-5p	MIMAT0004947	9.32	-1.79	4.48E-02	-0.21
hsa-miR-887-3p	MIMAT0004951	6.98	1.45	4.77E-02	0.60
hsa-miR-95-3p	MIMAT0000094	9.67	-1.73	2.30E-02	-0.18

Supplementary Table 2. List of differentially expressed miRNAs in FAPs *versus* MPs. The table indicates the Log2 Base Mean in FAPs and MPs, the Log2 Ratio between FAP and MPs, the adjusted p.value and the modulation by TGFβ1 in each cells. Thresholds used for selection of the miRNA candidates are log2 Base Mean>7, Log2 FC>2 and adj.Pval <0.05.

Name	log2 BaseMean FAP	log2 BaseMean MP	log2FC FAP vs MP	padj FAP vs MP	TGF-β effect on FAPs	TGF-β effect on MPs
hsa-miR-1	5.96	13.48	-10.31	8.47E-23	None	DOWN
hsa-miR-10a-5p	8.86	13.15	-2.85	1.77E-05	None	None
hsa-miR-128-3p	11.39	13.44	-2.93	2.76E-04	None	DOWN
hsa-miR-133a-3p	8.78	16.33	-10.38	4.69E-12	None	DOWN
hsa-miR-133a-5p	1.03	7.70	-9.22	1.98E-15	None	DOWN
hsa-miR-133b	7.43	15.58	-10.59	1.90E-09	None	DOWN
hsa-miR-138-1-3p	8.53	5.03	3.86	4.29E-03	None	None
hsa-miR-138-5p	13.28	10.70	2.74	1.96E-04	None	None
hsa-miR-139-5p	4.25	8.95	-6.87	1.32E-10	None	DOWN
hsa-miR-143-3p	15.89	11.82	3.59	7.11E-06	UP	None
hsa-miR-145-3p	10.60	6.25	3.61	2.03E-03	UP	None
hsa-miR-146b-5p	12.04	9.56	3.66	1.01E-06	DOWN	None
hsa-miR-148a-3p	11.93	8.46	4.03	5.95E-08	None	UP
hsa-miR-148a-5p	7.61	3.64	4.46	1.52E-05	None	None
hsa-miR-181c-5p	4.92	7.91	-3.08	1.75E-03	None	None
hsa-miR-188-5p	7.24	10.62	-4.41	1.31E-06	None	DOWN
hsa-miR-193a-5p	11.83	10.39	2.12	7.33E-03	None	None
hsa-miR-199a-3p	17.22	15.01	2.75	5.60E-07	UP	UP
hsa-miR-199a-5p	16.07	13.81	2.44	2.54E-05	UP	UP
hsa-miR-206	7.71	16.59	-10.44	5.36E-18	None	DOWN
hsa-miR-214-3p	14.96	12.90	2.13	4.90E-02	UP	UP
hsa-miR-214-5p	12.36	10.15	2.37	1.33E-03	UP	UP
hsa-miR-218-5p	10.74	7.64	4.35	5.68E-06	DOWN	None
hsa-miR-224-3p	9.17	5.89	3.99	1.46E-05	None	None
hsa-miR-224-5p	12.31	9.30	3.63	8.74E-07	None	None
hsa-miR-23b-5p	5.28	8.04	-2.94	3.31E-03	None	None
hsa-miR-24-1-5p	5.97	8.94	-3.06	1.10E-03	None	None
hsa-miR-27b-5p	5.69	8.58	-2.97	1.87E-03	None	None
hsa-miR-31-3p	13.36	11.71	2.50	2.69E-04	None	UP
hsa-miR-31-5p	17.18	15.29	2.46	1.46E-05	None	UP
hsa-miR-362-3p	5.47	8.01	-3.27	2.66E-03	None	DOWN
hsa-miR-362-5p	9.48	11.47	-2.86	1.05E-04	None	DOWN
hsa-miR-376a-3p	9.89	11.15	-2.00	2.07E-02	None	None
hsa-miR-376b-3p	9.78	11.31	-2.23	7.33E-03	None	None

hsa-miR-410-3p	8.89	10.41	-2.69	1.06E-03	None	None
hsa-miR-424-5p	10.67	12.93	-2.27	1.90E-02	None	None
hsa-miR-4301	11.15	10.31	2.26	1.08E-02	None	UP
hsa-miR-433-3p	7.30	9.22	-3.25	2.35E-04	None	DOWN
hsa-miR-450a-5p	10.60	13.45	-2.86	6.65E-04	None	None
hsa-miR-450b-5p	5.21	8.68	-3.41	3.49E-04	None	None
hsa-miR-452-3p	9.57	7.28	3.36	1.42E-04	DOWN	None
hsa-miR-452-5p	11.26	8.27	3.76	2.31E-06	DOWN	None
hsa-miR-483-3p	8.52	11.82	-3.07	8.01E-05	UP	None
hsa-miR-483-5p	7.13	10.87	-3.61	1.23E-05	UP	None
hsa-miR-486-5p	5.59	10.29	-5.48	1.75E-03	None	DOWN
hsa-miR-487a-3p	6.27	8.08	-3.01	2.03E-03	None	None
hsa-miR-499a-5p	6.22	10.90	-7.74	2.65E-14	None	DOWN
hsa-miR-500a-3p	7.96	9.49	-2.56	2.20E-02	None	DOWN
hsa-miR-500a-5p	8.75	10.88	-3.10	4.17E-05	None	DOWN
hsa-miR-500b-5p	7.47	9.01	-2.36	9.26E-03	None	DOWN
hsa-miR-501-3p	6.20	8.05	-2.60	7.57E-03	None	DOWN
hsa-miR-501-5p	6.99	10.55	-4.82	1.29E-07	None	DOWN
hsa-miR-502-5p	6.13	8.50	-2.98	1.68E-03	None	DOWN
hsa-miR-503-3p	5.15	7.87	-3.87	1.09E-04	None	None
hsa-miR-503-5p	6.69	9.30	-3.31	2.07E-02	UP	None
hsa-miR-532-3p	8.84	10.68	-2.68	1.18E-02	None	DOWN
hsa-miR-532-5p	10.46	12.54	-2.98	1.52E-05	None	DOWN
hsa-miR-542-3p	7.68	11.17	-3.40	2.04E-02	None	None
hsa-miR-660-5p	10.67	12.91	-3.01	7.67E-06	None	DOWN
hsa-miR-675-3p	4.07	9.97	-6.53	8.70E-06	None	DOWN
hsa-miR-675-5p	6.46	12.85	-7.87	3.54E-23	UP	DOWN
hsa-miR-877-5p	5.10	7.47	-2.24	3.88E-02	None	None

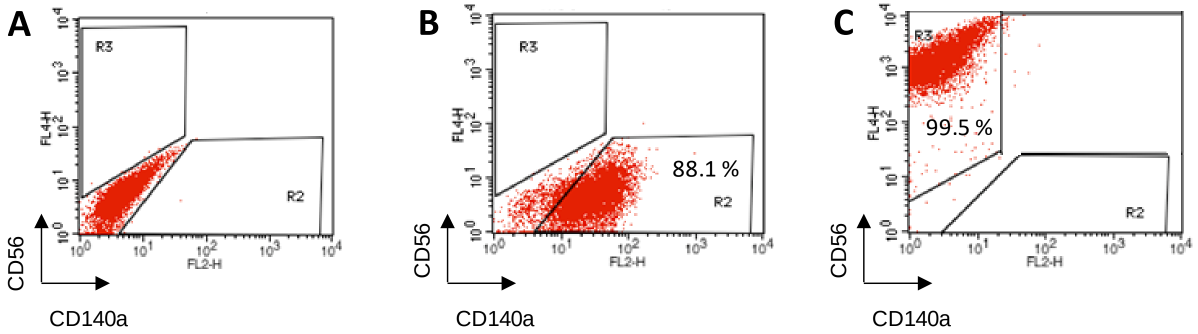

Figure S1: Immunophenotype controls showing the purity of FAP and MP populations performed by flow cytometry. FAPs and MPs were routinely checked with CD56 and CD140a markers by FACS. (A) CD140 isotype was used as negative control for the flow cytometry experiments. (B) FAPs showed more than 88% of PDGFRα positive cells (CD140a) and no CD56 positive cells. (C) MPs were characterized by up to 99% of CD56 positive cells (NCAM expression) and no PDGFRα positive cells (CD140). The images were representative for all samples.

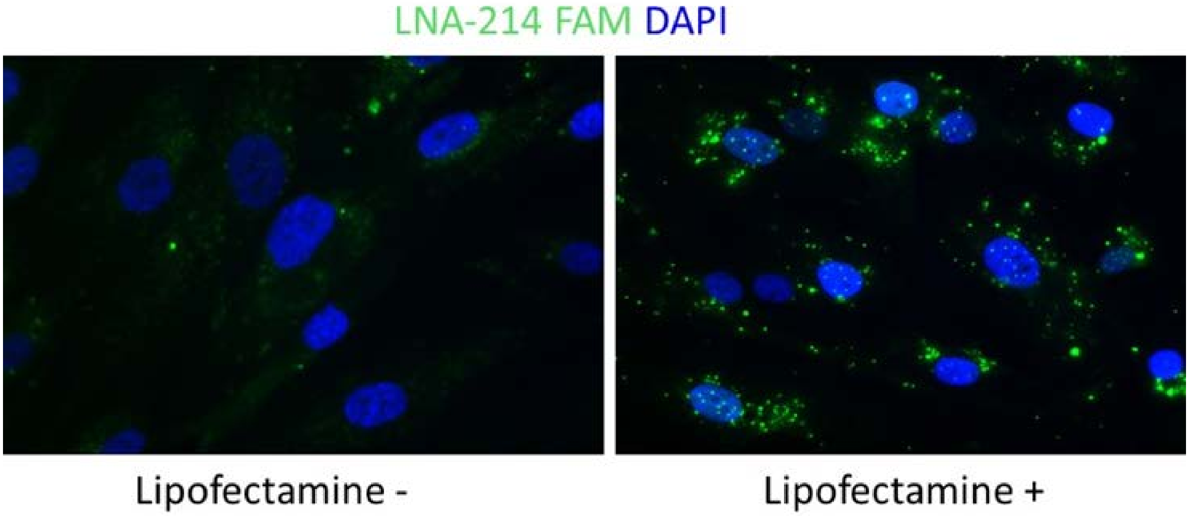

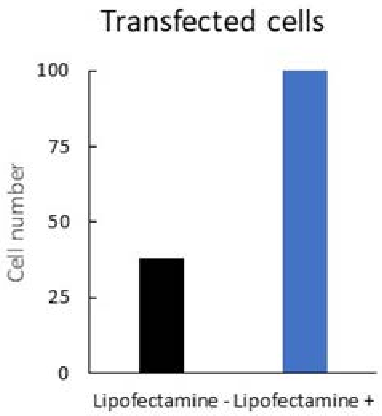

Figure S2: Visualization and quantification of fluorescent LNA-214-4p-FAM in FAPs. FAPs were treated with LNA-214-4p-FAM (FAM, green) in the presence or the absence of Lipofectamine (RNAi MAX™) as described in the Materials and Methods section. Nuclei were labeled with DAPI (blue).

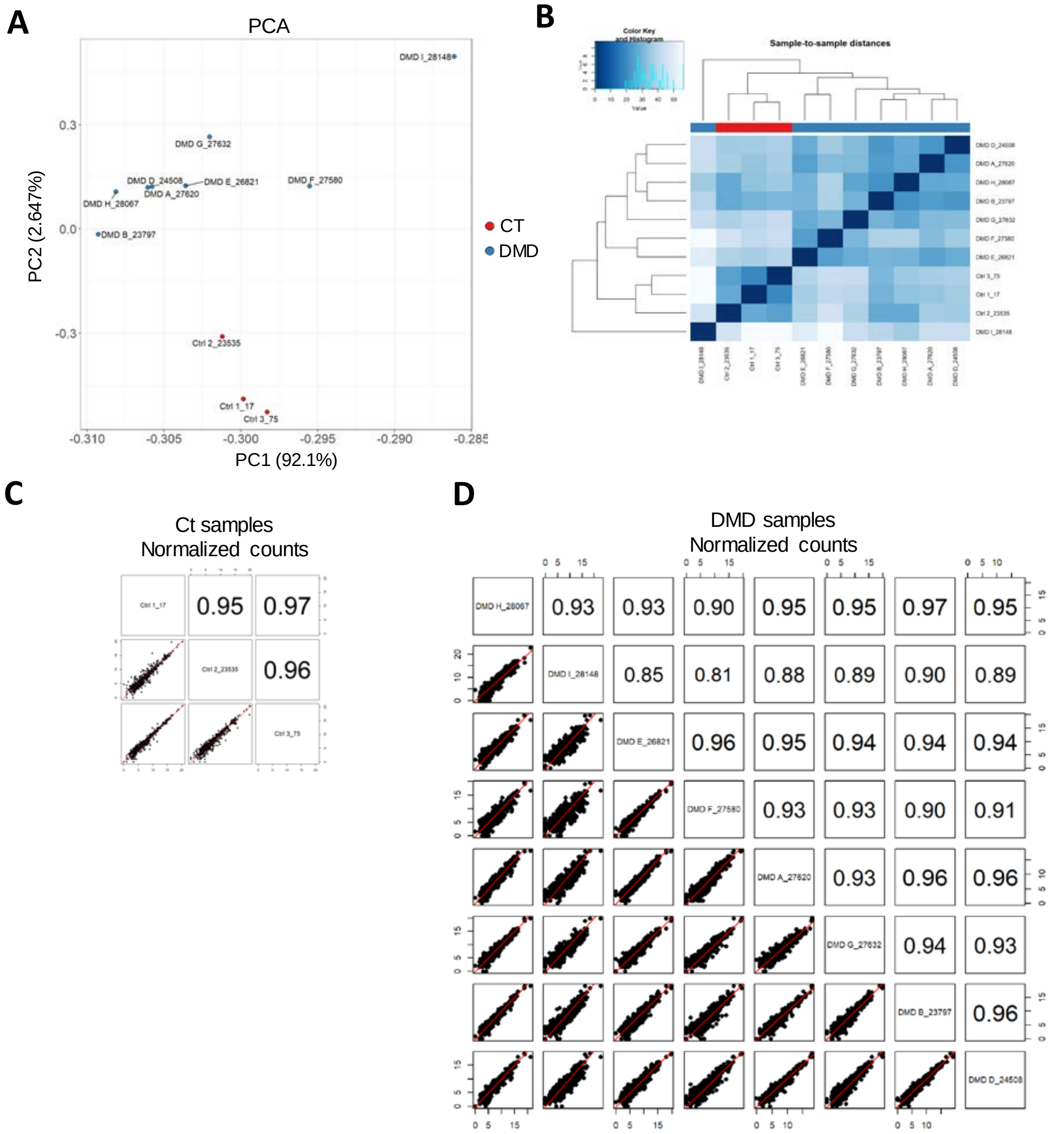

Figure S3: Overview of miRNAs data expression in DMD versus control muscle biopsies. (A) Principal component analysis of 8 DMD muscle biopsies and 3 age-matching control muscle biopsies using miRNA normalized counts. **(B)** Heatmap showing the Euclidean distances between the samples based on their respective miRNAs expression profiles. (C, D) Pairwise correlation between control samples **(C)** and DMD samples **(D)** respectively. The left diagonal corresponds to a scatterplot of the two variables above it and to its right; the right diagonal prints the correlation coefficient for the same variables.

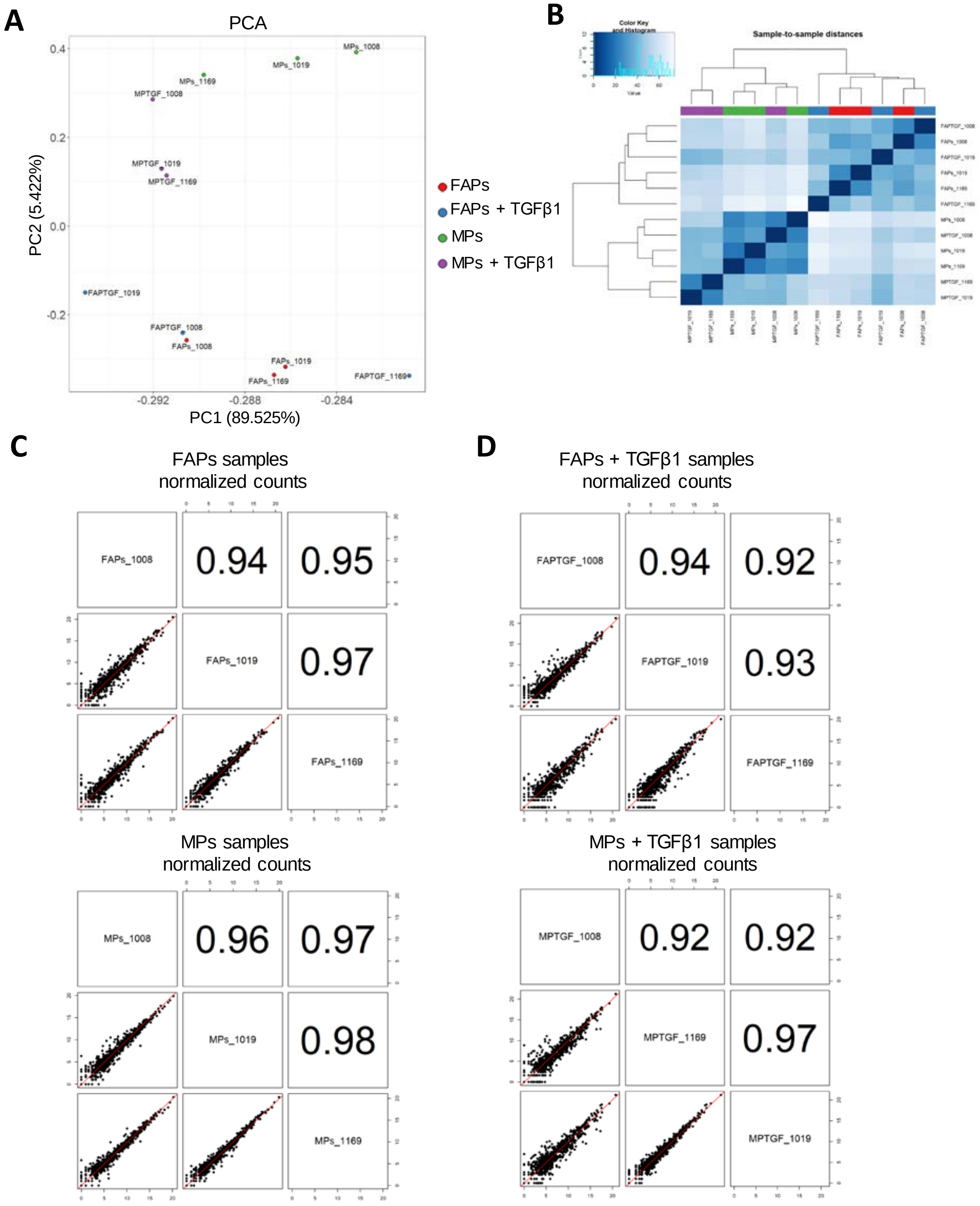

Figure S4: Overview of miRNA expression data in control and TGFβ1-treated FAPs and MPs. (A) Principal component analysis of control and TGFβ1 stimulated FAPs and MPs samples using miRNA normalized counts. **(B)** Heatmap showing the Euclidean distances between the samples based on their respective miRNAs expression profiles. **(C, D)** Pairwise correlation between control FAPs and MPs samples **(C)** and TGFβ1 stimulated FAPs and MPs **(D)** samples respectively. The left diagonal corresponds to a scatterplot of the two variables above it and to its right; the right diagonal prints the correlation coefficient for the same variables.

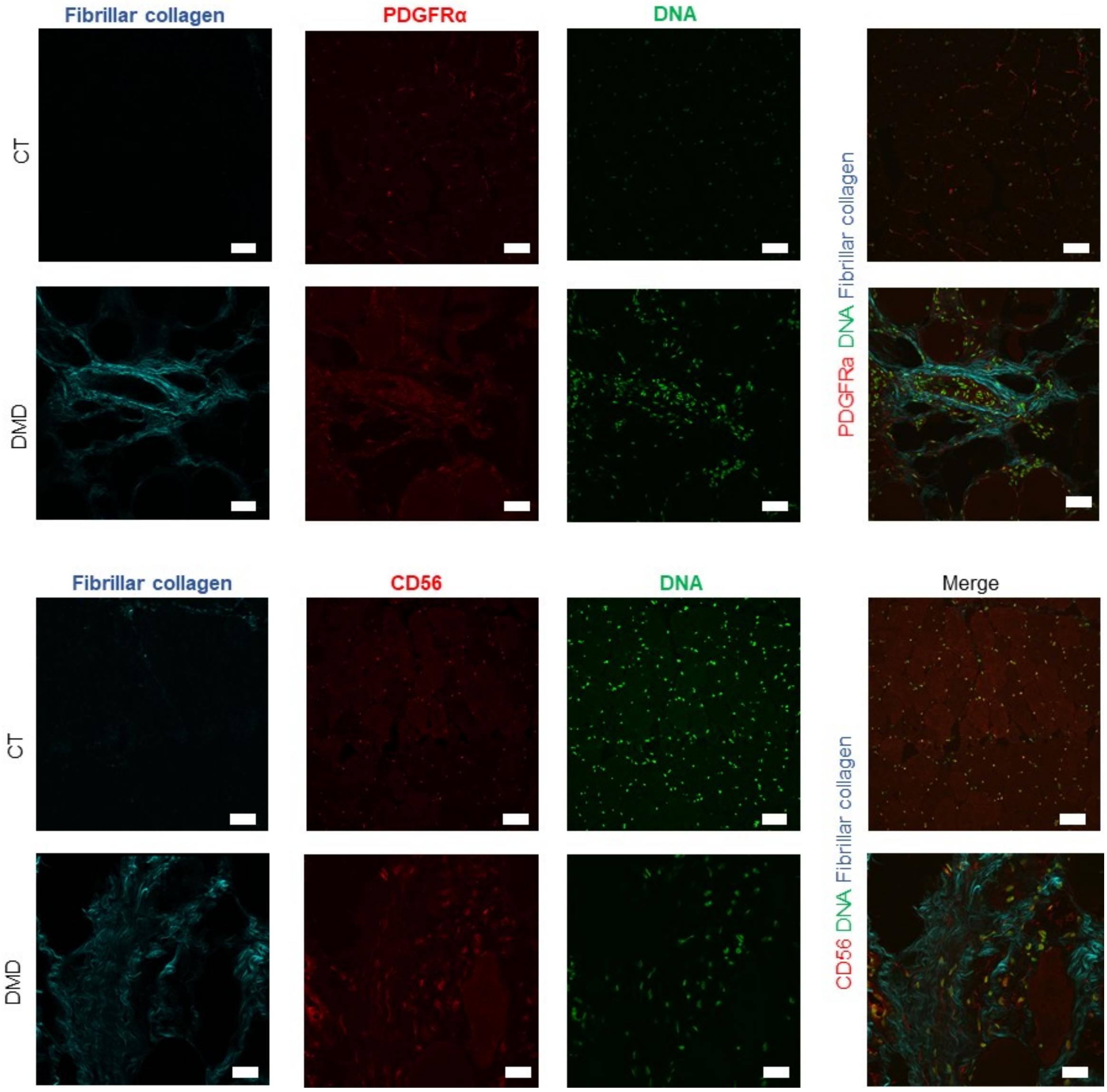

Figure S5: Collagen, CD56 and PDGFRα visualization in DMD and healthy control samples. Single channel and merge of intramuscular fibrillar collagen was visualized in cyan by second-harmonic generation imaging on cryostat sections from one healthy (CT) or DMD donor. Section were co-stained with CD56 (red) or PDGFRα (red) and DRAQ5 (green). Representative images of staining in healthy or DMD donor were shown. Scale bar shows 50 µm.

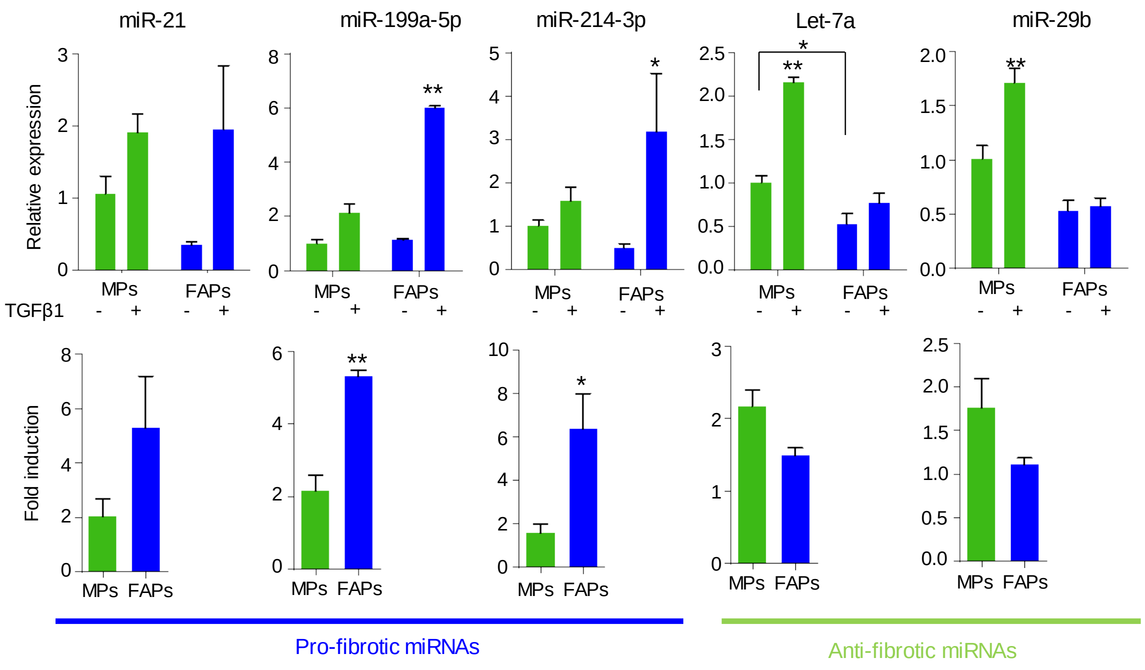

Figure S6. Expression and regulation of a subset of pro- and anti-fibrotic miRNAs in MPs and FAPs stimulated or not with TGFβ1 (5 ng/mL) for 48 hours. FAP and MP cells are described in table 1 (1008, 1019, 169). RNU44 was used as reference gene. Data are expressed as mean ± SEM (n=3). * $p<0.05$ and ** $p<0.01$ compared to the non-stimulated condition.

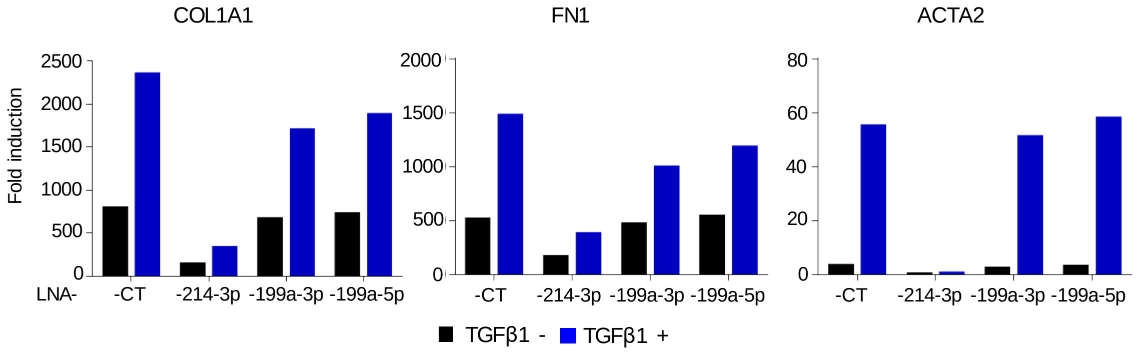

Figure S7. Effect of of miR-199a-3p, miR-199a-5p and miR-214-3p knockdown on FAP differentiation. Relative expression of fibrotic markers *COL1A1*, *FN1* and *ACTA2* in FAPs transfected with LNA-CT, LNA-199a-3p, LNA-199a-5p or LNA-214 for 24 hours, then cultured with differentiation medium alone or supplemented with 1.5 ng/mL of TGFβ1 for 2 days. *TBP* was used as housekeeping gene (n=1).

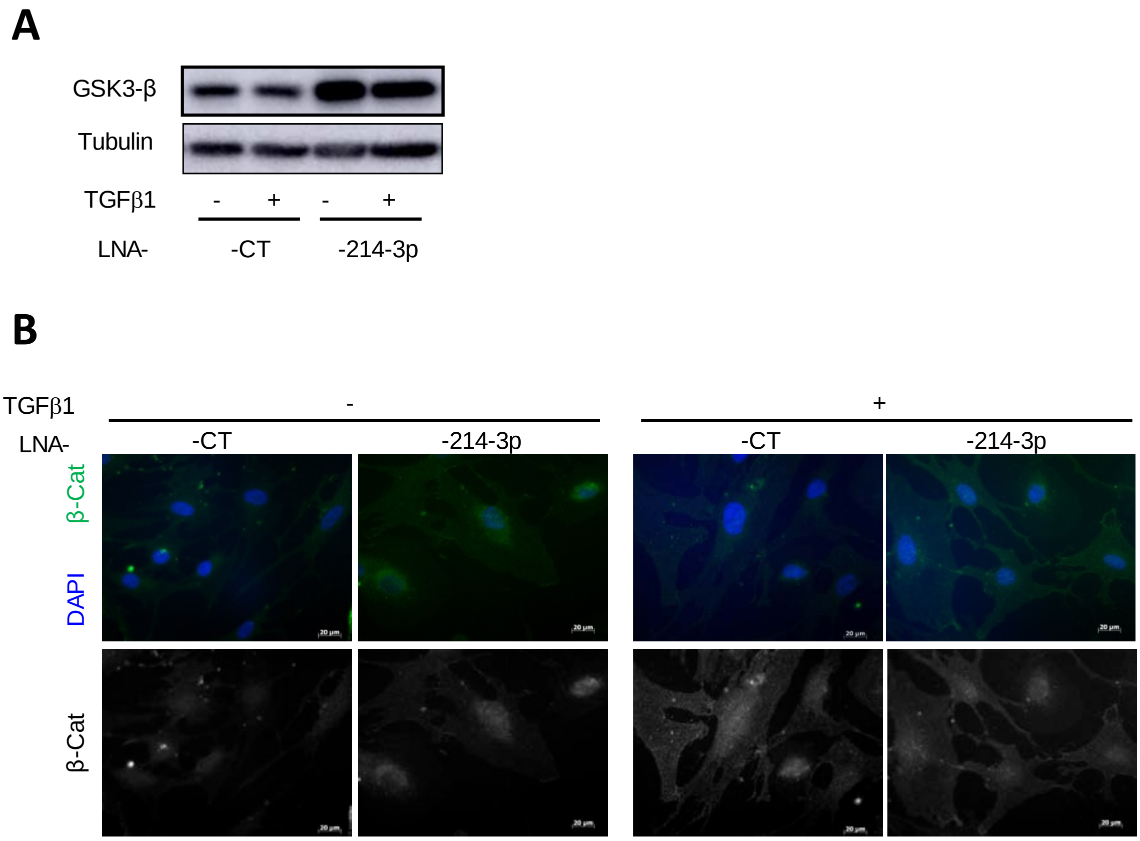

Figure S8. Impact of miR-214-3p on GSK3-β / β-Catenin axis. (**A**) GSK3-β protein was detected by immunoblot in FAPs transfected with LNA-CT or LNA-214-3p and then stimulated or not with TGFβ1 for 2 days. Tubulin was used as loading control. One of three independent replicates has been shown. (**B**) Intracellular β-Catenin localization evaluated by immunofluorescence in FAPs transfected with LNA-CT or LNA-214-3p and then stimulated or not with 1.5 ng/mL of TGFβ1 for 2 days. DAPI was used as nuclear staining. One of three independent replicates has been shown.
